# Using TECHnology to predict the future of biomedical education

**DOI:** 10.1002/2211-5463.13953

**Published:** 2024-12-12

**Authors:** Robert A. Harris, Hasan Kazdağlı

**Affiliations:** ^1^ Department of Clinical Neuroscience Karolinska Institutet, Centre for Molecular Medicine, Karolinska Hospital Stockholm Sweden; ^2^ Vocational School of Health Services İzmir University of Economics İzmir Türkiye

**Keywords:** artificial intelligence, digitilization, education, research

## Abstract

Biomedical research is currently benefiting from a technological revolution in which multiple forms of omics are permitting unprecedented characterization of molecular pathways. Likewise, medical device and Ai‐assisted technologies now make diagnoses and medical imaging more accurate. The field of education is also starting to embrace how technology can promote pedagogical development and student learning. But how will this landscape look like in 2050? With the premise that collaboration will be central to teaching and learning practices, that Together, Everything Can Happen (TECH), we examine the emerging trends and innovations in biomedical education, exploring how they will influence the field's evolution and shape future teaching practices in the coming years.

## Graphical abstract

We examine the emerging trends and innovations of Ai/digitalization in biomedical research and education, exploring how they will influence the field's evolution and shape future teaching practices in the coming years.

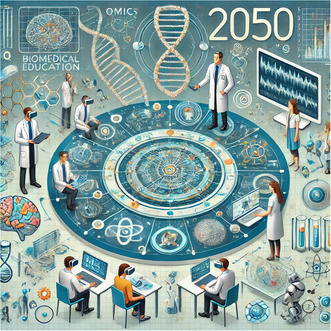

**Abbreviations**
Ai: Artificial Intelligence
AR: augmented reality
HUPO: Human Proteome Organization
IPE: interprofessional education
MR: mixed reality
SOLO: Structure of the Observed Learning Outcome
TECH: Together, Everything Can Happen
VR: virtual reality

## A university is a hub of R&D

The remit of a university is research and education, and a doctoral education is the fusion of these two areas, which are often otherwise considered separately. Both research and education have undergone significant development over time, with each era bringing its own technological and cultural advancements. While the last two decades have seen a rapid acceleration in these developments—particularly with the rise of the omics revolution, Big Data, and digital collaboration—the foundation for these transformations was laid by earlier innovations. These advancements have collectively provided us with unprecedented possibilities to teach new generations of scientists across a variety of disciplines, opening the door to unparalleled joint ventures on a global scale.

## XYZ‐disciplinarity as a basis for development

The classic disciplines of mathematics, chemistry, physics, and biology remain foundational as stand‐alone undergraduate subjects, but today's educational landscape increasingly favors inter‐, intra‐, and multi‐disciplinary approaches. Fields such as genomics, proteomics, metabolomics, computing and digital technologies are now integral to the evolving curriculum. While the classical syllabi are still relevant, the additional layers of complexity and detail—growing each year—must be integrated into our teaching. Collaboration and multi/interdisciplinary approaches, which have been well‐documented in foundational reports such as the 2010 AAAS ‘Vision and Change in Biology Education’ and the 2009 HHMI ‘Scientific Foundations for Future Physicians,’ have become the norm in modern research [1, 2]. The relevance of the 2009 and 2010 reports is still significant even after 15 years, as the fundamental need for collaborative science and interdisciplinary education has only intensified. The increasing complexity of scientific challenges, particularly in areas such as systems biology, precision medicine, and climate science, requires not just collaboration but deeper integration across disciplines [3, 4]. These reports laid the groundwork for modern educational reforms that continue to push the boundaries of how we train future scientists to navigate and solve these intricate challenges.

These principles are evident in the growing trend towards multi‐authorship in top‐tier journal publications, particularly in fields such as genomics, where large‐scale studies often require contributions from multiple institutions and specialties [5]. For example, the Human Genome Project involved thousands of researchers from across the globe and resulted in numerous multi‐authored papers in journals such as *Nature* and *Science*. Similarly, collaborative efforts in the field of proteomics which rely on multi‐disciplinary teams to interpret vast datasets have yielded high‐impact publications, such as those by the Human Proteome Organization (HUPO) [6, 7, 8].

## 21st century skills

Collaboration (as shared conceptualization) is one of the so‐called *21st Century Skills* first proposed by the US National Research Council (USNRC) in 2012 [9], along with Creativity (unexpected and appropriate), Critical Thinking (solving problems, making decisions, learning new concepts), and Communication (including multiple digital media). Importantly, these ‘4C’ skills also include digitalization as a technical aspect. These are mirrored in the OECD Learning Framework 2030 [10] which also includes a synthesis of (i) Cognitive and meta‐cognitive skills (including critical thinking), (ii) Social & emotional skills (including collaboration), and (iii) Practical & physical skills (including communication technologies). It is through a combination of these diverse skill sets that students are predicted to have the best possibility to be innovative.

The integration of collaborative practices into educational curricula is supported by various pedagogical frameworks. For instance, interprofessional education (IPE) has emerged as a vital approach in health professional education, where students from different disciplines learn together, fostering an understanding of collaborative practice [11]. This model not only prepares students for future teamwork but also addresses the limitations of traditional, uniprofessional educational approaches that can hinder effective collaboration in professional settings [12]. Additionally, the 4C model of collaboration highlights the importance of diverse skill sets and the need for students to engage in collaborative work to solve complex problems [13]. Such frameworks underscore the necessity of preparing students for collaborative environments, which are increasingly prevalent in modern workplaces.

Moreover, the role of technology in facilitating collaboration cannot be overlooked. The advent of Web 2.0 technologies has transformed collaborative learning by providing platforms that support interactive and participatory learning experiences [14]. These technologies enable students to engage in collaborative reasoning and problem‐solving, thereby enhancing their learning outcomes and preparing them for the demands of the digital age [15]. Virtual collaboration skills are also critical, as many contemporary work environments require effective teamwork across geographical boundaries [16].

While many educational institutions have embraced technology and provide collaborative tools to both staff and students, they must also incorporate training that equips students with the necessary skills to collaborate effectively in both physical and virtual contexts. For example, students must be adept at using digital communication platforms, managing online projects, and navigating collaborative software efficiently [16]. In addition, soft skills such as digital etiquette, virtual leadership and intercultural communication are becoming increasingly important as remote and hybrid work environments become the norm [17, 18]. Fostering these competencies will not only enhance students' ability to collaborate but will also align with the broader digital transformation of research and academia, where virtual teamwork and digital tools play a pivotal role in driving innovation and scholarly collaboration.

## The digital transformation of research

Digitalization is nothing new to research *per se*—there have been image analysis program softwares available for years. However, with the recent development of Artificial Intelligence (Ai) machine learning programs [19, 20], there are many fields of research that have been significantly transformed by the increased complexity of pattern recognition, predictive analysis and rapid data processing that Ai technologies provide. Drug discovery platforms currently use Ai to identify new precise and personalized drugs, such as Berg's pancreatic cancer drug BPM31510 [21] or Pfizer's IBM Watson platform for immuno‐oncology drugs [22]. DeepMind's AlphaFold has revolutionized structural biology [23] and be it chemical reactions (Reactome [24]) or cell morphology (Deep Cell's Morpholome [25]) that is of interest, there are Ais to accommodate everyone's wishes (see Table 1 for more examples). Maybe most significantly, deep learning approaches such as Stratipath's breast cancer diagnosis system is now more reliable than human diagnoses [26], and so the field of personalized medicine will undoubtedly continue to be transformed by implementation of Ai in many clinical situations. While the advantages of Ai in research will be obvious from these few examples, the challenge is how to educate researchers into their appropriate use.

**Table 1:** Commonly used digital tools for research, visualization and collaboration.

Virtual reality	Labster, Onlabs, Confocal VR, BioVR (Virtual Reality for biological visualization)
Molecular visualization	UCSF ChimeraX, Nanome, Narupa (Molecular dynamics), Jmol (Molecular structures visualization)
3D modeling software	Turbosquid, Sketchfab, Maya, Cinema 4D, Blender (These are general‐purpose 3D modeling tools, not specific to any particular discipline)
Drug discovery	PhenomBeta (Deep learning for drug discovery), Galactic
Research tools	Perplexity, Research Rabbit, Connect Papers, Inciteful, Cite (AI‐powered tools for literature reviews and research insights)

## Digitalization in education

Apart from accelerating research development, there has been a similar significant digitalization of educational practices during the past decades [27]. Educational digitalization refers to the integration of various technologies into teaching and learning environments to enhance educational outcomes. These include Artificial Intelligence (AI), Virtual Reality (VR), Augmented Reality (AR), Mixed Reality (MR) and digital platforms for content delivery and communication. These technologies, while often discussed together, serve distinct purposes in the educational landscape.

VR and AR are becoming common in modern teaching, offering immersive experiences that supplement traditional methods [28]. The recent emergence of MR which combines these approaches provides hitherto unprecedented opportunities for interactive experiences. For instance, while a virtual post‐mortem cannot fully replace the hands‐on experience of medical training, repeated digital simulations for specific surgeries provide valuable practice and have become standard in many hospitals. Furthermore, some medical programs now combine medical training with technology education, fostering physicians who are adept with digital tools.

Digital platforms, such as mobile learning solutions, also play a crucial role in modern education. These platforms facilitate communication, content delivery and collaboration between students and educators. Tools such as *WeChat* and *PowerPoint*, integrated into platforms like the *Rain Classroom*, provide a ‘one‐stop’ mobile teaching solution widely used in Chinese classrooms [29]. Unlike Ai, which focuses on data‐driven personalization, digital platforms provide a flexible infrastructure for learning activities and communication.

Artificial Intelligence (Ai) has introduced several new possibilities in education. Some of the key benefits of Ai include:

- Personalized learning: Ai can track student progress and adjust the pace of instruction accordingly.
- Personalized content: Ai can generate customized modules tailored to each student's learning needs.
- Automatization of administration: Ai tools can streamline processes such as admissions and examination management.
- Educational data analysis: Ai helps educators identify trends and patterns in student performance, facilitating data‐driven teaching decisions.

**Fig. 1:**
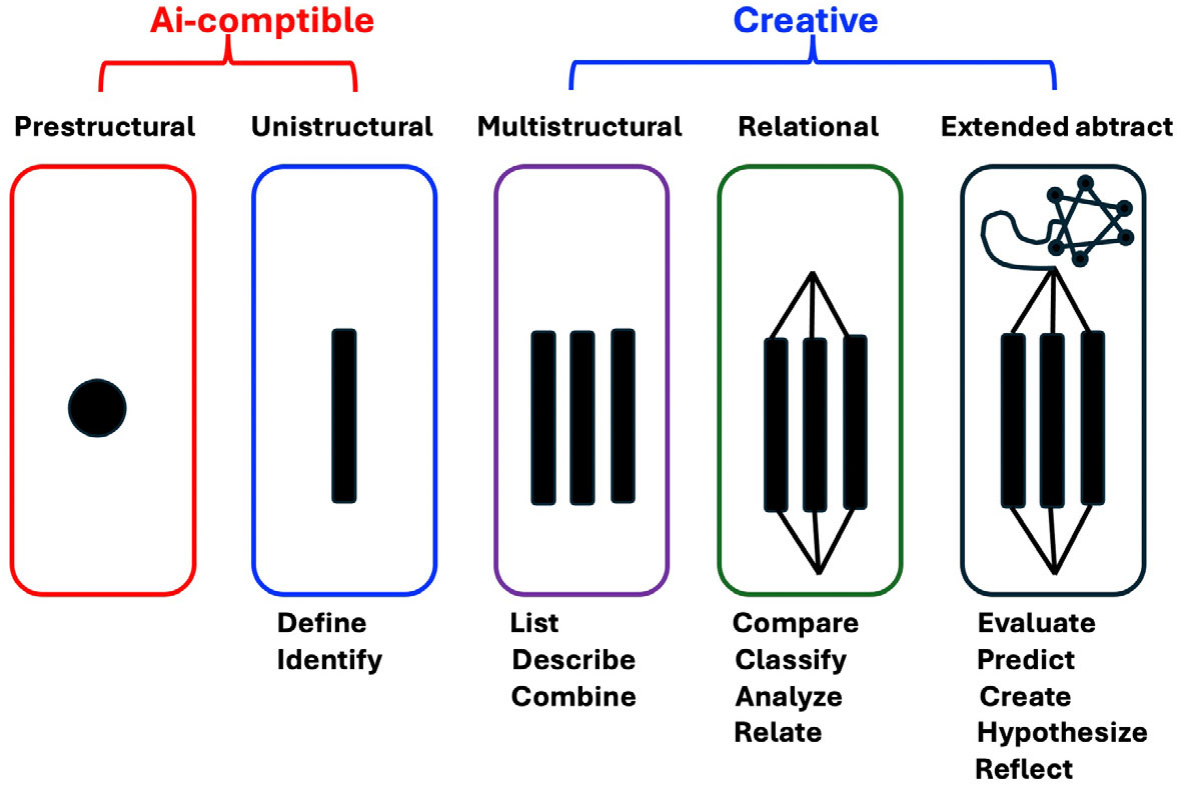
Biggs' SOLO taxonomy of learning applied to digitalization.

While the benefit of reduced human administrator costs is clear with digital admissions platforms like *Slate*, widely used in North America [30], Ai's impact on educational assessments has also raised concerns. The advent of large language models like ChatGPT forces educators to rethink how they assess student knowledge. Ai tools excel at managing lower‐order cognitive tasks, such as recalling factual information, but they struggle with tasks that require higher‐order thinking and individual creativity. Applied to Biggs' SOLO taxonomy of learning, Ai could effectively support Prestructural, Unistructural, and Multistructural levels, but will face challenges in handling the Relational and Extended Abstract levels [31] (see Fig. 1).

## Co‐creation in education: The ‘Produser’ model

Another important aspect of educational digitalization is the ‘Produser’ model, a concept first described by Axel Bruns [32]. In this model, students and teachers co‐create personalized syllabi together, blurring the line between consumer and producer of educational content. This model fosters both traditional mentoring from educator to student and reverse mentoring, where students help educators adapt to new digital tools. Given that younger generations are growing up with digital technologies, they may become key facilitators of digital literacy among older educators when they enter university.

## Personalized and flexible learning

Digitalization in education also enables personalized and flexible learning experiences. Smart campuses use digital tools to tailor learning environments to the diverse needs of students [33]. This personalization not only enhances student engagement but also supports various learning styles, promoting inclusivity [34]. The COVID‐19 pandemic accelerated the adoption of digital technologies, institutionalizing their role in education and prompting a paradigm shift towards online and blended learning models [35]. This shift underscored the necessity for educators to develop technological pedagogical knowledge (TPK), allowing them to integrate digital tools effectively into their teaching practices [36].

## Future of digitalized education

Looking forward, this will drive even more personalized learning experiences, with data‐driven insights guiding individualized learning pathways. This will enable educators to cater to each student's unique needs and pace, fostering a more effective and engaging educational experience. Additionally, global connectivity could break down geographic and socio‐economic barriers, providing quality education to students in the most remote or underserved areas [37]. Blockchain technology could also be employed to verify credentials universally. Educators will likely benefit from continuous professional development delivered through AI‐driven platforms, which offer tailored training based on teaching practices and student outcomes.

While the digitalization of education provides a great many new opportunities, it is not without challenges [38]. The first is equity, as these digital technologies are not necessarily available to everyone. The second and more important challenge is that as the younger generations will have great knowledge about digital technologies, how will the older generations keep up with this? The third is the increased accessibility 3A aspect—access, anytime, anywhere. Is this what educators will wish for? Finally, the importance of social presence in online learning cannot be overstated. Almahdi *et al*. [39] argue that fostering a sense of community among online learners is essential, especially in light of the isolation experienced during the pandemic. This social dimension is critical for maintaining student motivation and engagement, and it helps mitigate the feelings of disconnection that can arise in fully online settings.

## Reconsidering the flipped learning model for the future

The flipped learning model, which has gained widespread attention in recent years, represents a significant shift from traditional lecture‐based instruction [40]. By allowing students to engage with instructional materials outside of the classroom and using in‐class time for interactive activities, the model aims to deepen learning through active participation [41]. However, as education continues to evolve, it is essential to question whether the traditional flipped model fully aligns with the future needs of learners.

The future of education may require a more flexible approach that integrates both online and offline learning experiences to meet the diverse needs of students. Personalized learning paths, adaptive learning technologies, and a blend of digital and face‐to‐face instruction could offer more tailored and responsive learning environments. This would allow students to progress at their own pace while benefiting from interactive, collaborative and customized experiences in the classroom.

It is important to consider that a reliance on technology in the flipped model might widen the gap for students who lack access to digital tools or reliable internet. To promote equity and inclusivity, future learning models must balance online components with robust offline alternatives, offering flexible and adaptable structures that cater to diverse student needs. The flipped model may need adjustments, such as incorporating real‐time feedback and personalized content, in order to remain relevant and effective in addressing the educational challenges of the future.

## Development of educator competencies

The digital transformation of education is fundamentally reshaping the competencies required of educators, necessitating a comprehensive understanding of digital literacy and pedagogical strategies that leverage technology effectively. As educational institutions transition towards a more digitally integrated framework, it becomes imperative to focus on developing the digital competencies of educators to enhance teaching and learning outcomes.

To effectively use a variety of forms of digital media to promote inquiry‐based learning, educators must develop both knowledge of and competence using digital tools, as well as the ability to link these tools to pedagogical strategies. Digital literacy refers to having a basic understanding and competency in using digital tools, while digital fluency refers to being adaptable and innovative in the digital world [42]. Educators will not only need to be literate, but fully fluent. This will require targeted training and lifelong learning in order to develop sufficient competence to keep pace with the continued development of Ai.

If educators save time by embracing digital and Ai technologies, this shift could open opportunities for practices previously not prioritized. For instance, the use of educational escape rooms to foster collaboration in solving subject‐specific puzzles, or the creation of innovative spaces such as the Invention Rooms at Imperial College London, where students and educators collaborate on creative projects [43].

Furthermore, the shift towards Education 4.0, a term that refers to the fourth industrial revolution's influence on education, emphasizes the need for educators to develop a blend of technical, social, and cognitive skills. Education 4.0 is characterized by personalized learning, the use of smart technologies, and an emphasis on creativity and critical thinking. The TADEO Method, which integrates technology into teaching practices, equips students and educators with essential skills for effective communication and collaboration in this digital context [44]. This approach enhances the learning experience and prepares educators to navigate the complexities of modern educational demands.

In this digital future, where Ai and digital tools may take on more roles in information dissemination, the flipped classroom model could become increasingly prevalent [40]. In this model, educators would focus on deepening students' understanding through interactive activities such as discussions, Q&A sessions, and personalized feedback. This would position educators more as facilitators who help students connect and consolidate ideas, using life and historical experiences to impart wisdom in ways that Ai cannot (yet) replicate, particularly through ‘storytelling’. This evolution in teaching practices redefines the educator's role, emphasizing human elements that complement digital tools.

## Universities must prepare for the digital transformation

The digitalization of higher education is a transformative process that is reshaping universities' operational, pedagogical, and administrative frameworks. If universities will efficiently adapt to the ever‐developing modern academic world, it is clear that they will have to make strategic decisions for how to facilitate this transformation. These might include:

- Faculty development—modernization of teaching practices
- Investment in infrastructures
- Student support services for technologies
- New assessment and evaluation strategies
- Promote collaboration with external technology partners

It will follow from the previous discussion that the often more senior generations capable of making such decisions will also have to acquire sufficient knowledge of the possibilities in order to do this efficaciously.

The establishment of online education platforms represents a fundamental shift in how universities deliver educational content. Many higher education institutions (HEIs) have successfully digitalized their courses, making education more accessible and flexible for students [45]. This transition not only facilitates learning but also enhances the efficiency of educational management and research processes. The ability to offer courses online allows universities to reach a broader audience, including non‐traditional students who may not be able to attend in‐person classes due to geographical or personal constraints [46].

Moreover, the adoption of digital technologies is influencing the strategic direction of universities. Effective management of digital transformation strategies is crucial for HEIs to thrive in a competitive educational landscape [46]. This includes the integration of advanced technologies into the curriculum and administrative processes, which can lead to improved operational efficiency and enhanced student experiences. The shift towards a more technology‐driven approach necessitates that universities rethink their traditional models and embrace innovative practices that align with the digital age.

## Conclusion

### Look to the future in your crystal ball

As researchers we are used to postulating a hypothesis and devising ways to test this. A hypothesis here will be that the future development of research and education will be highly dependent on the continued development and sophistication of digital/Ai technologies, which will impact on not only research and educational practices, but also on how the administration is conducted. Wearable or implantable devices that record a wealth of biological information might provide 24/7 health monitoring that will provide ‘early‐warnings’ of ill‐health and stimulate a physical meeting with a real‐life doctor, as a compliment to online Ai‐directed health consultations that will be of sufficient standard to provide good medical advice for ‘normal’ health issues. Mixed Reality eyeglasses will not only enhance professional research and educational experiences but will also be a standard part of everyday life for applying digital information to e.g. shopping experiences or the real‐life enjoyment of foreign travels. The possibility of global collaboration within research and using more advanced digital and Ai platforms will allow comparative biological studies of human population data to a degree currently impossible, as long as legislation does not hinder this development. Virtual avatars of humans that not only resemble human form but also faithfully imitate voices and speech styles will be common forms of education, and in society might also be one way that we compensate for the loss of loved ones and retain the social connections that promote metal wellness. The future might also include personalized medical imaging with a cellular resolution far greater than is currently possible. Students might define their own curricula by choosing digital courses from a globally available portfolio and define their own individual and specialized training programs. These crystal ball images might currently be blurry, but they are certainly colorful.

A second hypothesis will be that the roles of student and educator will be metamorphosed into a collaborative venture, in which both will rely heavily on digital/Ai technologies for among other things the quality control of their respective performance. Other than contributing to this development at our own universities, it will be equally stimulating to observe this development at other universities. Hopefully the collaborative culture that we promote for our students (4C) will also be demonstrated through the collaborative sharing of ‘best practices’ between universities. The ‘tech’ in technology relates not only to data, instruments or digital platforms, but also to the notion that ‘Together, Everything Can Happen.’

## Conflict of interest

The authors declare no conflict of interest.

## Peer review

The peer review history for this article is available at https://www.webofscience.com/api/gateway/wos/peer‐review/10.1002/2211‐5463.13953.

## Author contributions

RAH conceived the idea, determined the overall framework of the manuscript, led the writing process, and critically reviewed the manuscript. HK contributed to the sections of 21st Century skills, Digitalization in education and Development of educator competencies, and reviewed the manuscript.
